# Network pharmacology and *in vivo* experiment-based strategy to investigate mechanisms of JingFangFuZiLiZhong formula for ulcerative colitis

**DOI:** 10.1080/07853890.2022.2095665

**Published:** 2022-11-16

**Authors:** Mengyuan Wang, Jianan Li, Yuzhang Yin, Liying Liu, Yifei Wang, Ying Qu, Yanqiu Hong, Shuangshuang Ji, Tao Zhang, Nan Wang, Jinlong Liu, Xu Cao, Xiaobin Zao, Shuxin Zhang

**Affiliations:** a Dongzhimen Hospital, Beijing University of Chinese Medicine, Beijing, China; b Beijing University of Chinese Medicine, Beijing, China; c CHINA-JAPAN friendship Hospital, Beijing, China; d Key Laboratory of Chinese Internal Medicine of Ministry of Education and Beijing, Dongzhimen Hospital, Beijing University of Chinese Medicine, Beijing, China

**Keywords:** JingFangFuZiLiZhong formula, network pharmacology, ulcerative colitis, bioinformatics

## Abstract

**Background:**

Ulcerative colitis (UC), a chronic inflammatory disease, often cause carcinogenesis, disability, and intestinal perforation. The JingFangFuZiLiZhong formula (JFFZLZ) shows a good effect against UC in the clinic. Hence, we aim to investigate the mechanisms between JFFZLZ and UC *via* network pharmacology data mining and *in vivo* experiments.

**Methods:**

We obtained active constituents and related targets from public databases. The overlapped genes between JFFZLZ and UC targets were further analysed by enrichment analysis. The active constituents and hub targets were used to construct molecule docking analysis. We finally screened out nine hub targets and their expressions were verified in the Gene Expression Omnibus database and UC rats’ colon tissues after JFFZLZ treatment.

**Results:**

The results implied that JFFZLZ mainly regulated signal transduction, metabolites production, and inflammation pathways. The expression of STAT3, CXCL8, IL6, CXCL12, TNF, TP53, and PTPN11 were both upregulated in colon tissues of UC patients and UC rats. While RELA, EGFR, and TP53 were downregulated in UC patients, but upregulated in UC rats. Furthermore, JFFZLZ could repair UC rats’ colon mucosal damage and promote the healing of ulcers *via* regulating the hub targets.

**Conclusion:**

These results elucidated that the anti-UC effect of JFFZLZ was closely related to the inhibition of inflammatory response, inhibition of oxidative stress, and repairing colon mucosal damage through different signal pathways. The findings could contribute to a better understanding of the regulation mechanisms in JFFZLZ against UC.

**Key messages**
- JFFZLZ could reduce the inflammatory infiltration and repair UC rats’ colon mucosal damage.
- Through the network pharmacology-based strategy and public database mining, we obtained the hub targets and key pathways between JFFZLF and UC.
- The mechanism of JFFZLZ against UC was inhibition of inflammatory response and oxidative stress by regulating the expression of the hub targets.

## Introduction

Ulcerative colitis (UC) is a chronic inflammatory disease with recurrent attacks and unknown aetiology, which has caused a heavy health burden around the world [1]. Typical symptoms of UC patients are rectal bleeding, abdominal pain, diarrhoea, and mucous purulent bloody stool [2]. UC usually results in disability, colorectal cancer (CRC), and haematological diseases [3–6]. Extensive research has shown that the pathogenesis of UC is associated with exogenous substances, genetics, mucosal and epithelial barrier, immune response, intestinal flora, environmental, diet, and other factors [7–10]. For now, the therapies of UC include biologic agents, corticosteroids, immunosuppressants, probiotics, faecal bacteria transplantation, and traditional Chinese medicine (TCM) [11–13].

There have been a considerable amount of TCM studies on treating UC, such as Rhubarb Paeony decoction [14], BaiTouWeng decoction [15], QingChangHuaShi formula [16], and SanHuangShuAi decoction [17]. These studies demonstrated the effectiveness and security of TCM against UC. Meanwhile, the JingFangFuZiLiZhong formula (JFFZLZ) is one of the TCM we used in the clinical treatment, which had a therapeutic effect against UC. The JFFZLZ is comprised of *Herba Schizonepetae* (JingJie), *Radix Saposhnikoviae* (FangFeng), *Radix Aconiti Lateralis Preparata* (FuZi), *Radix Ginseng* (RenShen), *Rhizoma Zingiberis* (GanJiang), *Radix Glycyrrhiizae* (GanCao), and *Rhizoma Atractylodis Macrocephalae* (BaiZhu). On the basis of our clinical experience and observation, the JFFZLZ could alleviate the clinical symptoms, suppress the inflammatory reaction, and regulate inflammatory factors of UC patients. From the perspective of TCM, the core theory of JFFZLZ is to regulate host immunity, reduce the inflammatory response, and improve clinical symptoms [18–20].

**Figure 1.**
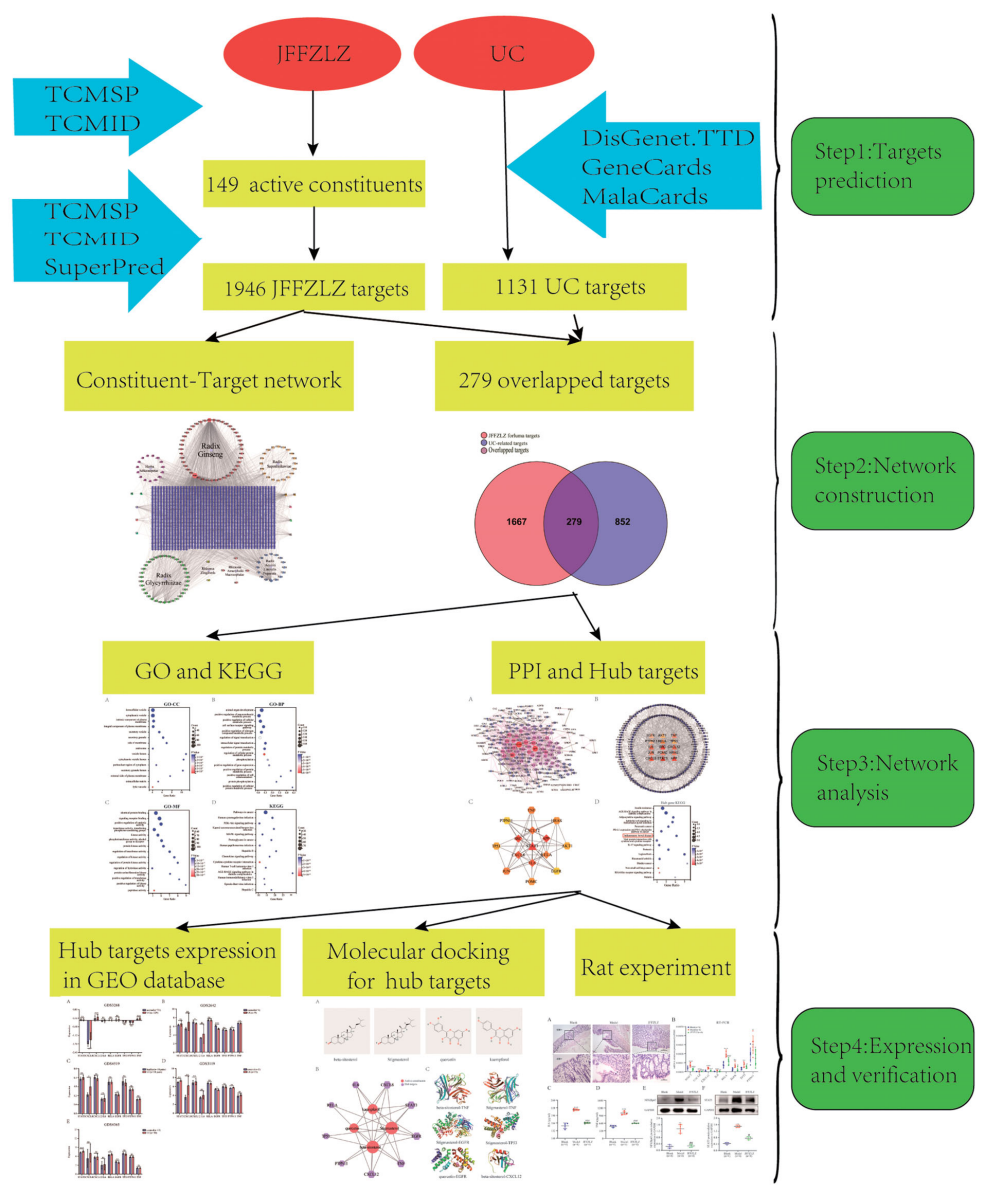
The flowchart of network pharmacology analysis of the mechanisms of JFFZLZ acting on UC.

Although the JFFZLZ has a good effect against UC, its potential pharmacological mechanisms are not fully elucidated. Meanwhile, the network pharmacology approach, also known as system pharmacology, has emerged as a powerful tool to explore the connection between drugs and diseases [21,22]. Therefore, in the current study, we aimed to investigate the key targets and regulatory mechanisms of JFFZLZ against UC with the help of network pharmacology and data mining methods. As shown in Figure 1, we first analysed the potential active constituents and putative targets of seven main herbs in JFFZLZ by TCMSP, TCMID, and SuperPred databases. Next, we screened disease targets of UC through the DisGeNET, GeneCards, MalaCards, and TTD databases. Then, we obtained the overlapped genes (OGEs) between JFFZLZ and UC targets to perform Gene Ontology (GO), Kyoto Encyclopaedia of Gene and Genomes (KEGG), and Protein-protein interaction (PPI) analysis, and ultimately obtained the hub targets. Next, we performed the molecular docking analysis between the common constituents and hub targets. After these analyses, the expressions of the hub targets were further detected in the Gene Expression Omnibus (GEO) datasets. Finally, we carried out an *in vivo* UC rat model with JFFZLZ treatment and detected the efficacy of JFFZLZ on UC rats by Haematoxylin–Eosin (HE) staining of colon tissues. We also analysed the hub targets expression in rats’ colon tissues by real-time quantitative polymerase chain reaction (RT-qPCR), ELISA, and Western blot (WB).

## Materials and methods

### Collection of potential active constituents in the JFFZLZ

The herbal formula of JingFangFuZiLiZhong formula (JFFZLZ) comprises seven traditional Chinese medicines, including *Herba Schizonepetae* (JingJie), *Radix Saposhnikoviae* (FangFeng), *Radix Aconiti Lateralis Preparata* (FuZi), *Radix Ginseng* (RenShen), *Rhizoma Zingiberis* (GanJiang), *Radix Glycyrrhiizae* (GanCao), and *Rhizoma Atractylodis Macrocephalae* (BaiZhu). The active constituents of each herb in JFFZLZ were harvested by use of the online databases TCMSP (http://tcmspw.com/tcmsp.php) and TCMID (http://www.megabionet.org/tcmid/). Oral bioavailability (OB) and Drug-likeness (DL) are used to filter active compounds [23]. The criteria for screening active constituents considering the characteristics in the TCMSP database is defined as OB≥ 30% and DL ≥ 0.18.

### Prediction of putative targets of JFFZLZ

The putative JFFZLZ targets were retrieved in TCMSP, TCMID, and SuperPred (http://prediction.charite.de) databases. After that, all proteins were converted into genes with the help of the Uniprot database (https://www.uniprot.org/) and acquired gene ID. Furthermore, the JFFZLZ constituent − target network was visualised through Cytoscape (version 3.7.2, https://cytoscape.org/index.html).

### Screening of candidate UC targets

Candidate UC targets were acquired from DisGeNET (http://www.disgenet.org/), GeneCards (https://www.genecards.org/), MalaCards (http://www.malacards.org/), and TTD (http://db.idrblab.net/ttd/) databases. The Keyword “*ulcerative colitis* (UC)” and “*Homo sapiens*” were used to search UC-related genes.

### Establishment of UC targets acted by the active JFFZLZ constituents

The Venny diagram (https://bioinfogp.cnb.csic.es/tools/venny/index.html) was constructed to visualise the overlapped genes (OGEs) between the JFFZLZ targets and UC targets.

### Establishment of GO and KEGG enrichment analysis

Gene Ontology (GO) function enrichment and Kyoto Encyclopaedia of Gene and Genome (KEGG) pathway enrichment are used to annotate functional characteristics and signal pathways involved in the targets [24]. The enrichment analysis of GO and KEGG was performed by the ClueGO plugin using OGEs. Value of *p* ≤ 0.01, GO Term Interval between three and eight, Cluster ≥ 3, and Kappa Score = 0.4 were defined as the criteria in the filtering terms of retrieval results in GO function enrichment. In the meantime, the filtering terms of retrieval results in KEGG were defined as the value of *p* ≤ 0.01, Cluster ≥ 20, and Kappa Score = 0.5. And, the top 15 data were selected as GO-CC, GO-BP, GO-MF, and KEGG based on the results of cluster, which were visualised in the form of bubble diagrams using Sangerbox tools (http://vip.sangerbox.com/home.html).

### Construction of PPI networks and screening of hub targets

The protein-protein interaction (PPI) network was derived based on the STRING database (https://string-db.org/cgi/input.pl). In this study, OGEs were inputted into the STRING database with confidence scores ≥ 0.7 as well as species limited to “*Homo sapiens*”. Then, PPI data were introduced into Cytoscape (version 3.7.2) to construct and visualise a PPI network, and the non-connection genes were removed. After obtaining the PPI network, the top 15 hub targets were acquired by the cytoHubba plugin of Cytoscape (version 3.7.2) according to the degree. Further, the top 15 genes were selected as hub targets based on the results of degree, and the PPI network in the top 15 genes was constructed by Cytoscape (version 3.7.2). Next, the enrichment analysis of KEGG in hub targets was performed by the ClueGO plugin using hub targets. And, the filtering terms of retrieval results in hub target KEGG were defined as the value of *p* ≤ 0.05, Cluster ≥ 3, and Kappa Score = 0.4. Bubble Diagrams were drawn by Sangerbox tools.

### Construction of molecular docking analysis in hub targets

Then, the common constituents and hub targets were used to construct a molecular docking network. The 3D structures of the hub target protein receptors were retrieved from the RCSB PDB database (https://www.rcsb.org) with species and resolutions limited to “*Homo sapiens*” and 2.0–3.0 °A. Moreover, the 2D structures of the common constituents were obtained from the TCMSP Database as ligands. Next, the molecular docking simulation was performed by AutoDockTools software (version 1.5.6), and the result was visualised in PyMOL software (version 2.2.0). The binding energy threshold condition ≤ −5.0 (BE, kcal/mol) was considered as the most favourable docking pose.

### Verification of the expression of hub targets

The expression of hub targets was verified in Gene Expression Omnibus (GEO) database (http://www.ncbi.nlm.gov/geo/), where the keywords were set as “*ulcerative colitis* (UC)”, “*Normal*”, and “*homo sapiens*”. The gene expression data for the analysis stem from the GDS3268, GDS2642, GDS3119, GDS4519, and GDS4365 datasets. For more, GDS3268 was composed of 129 patients with UC and 73 people with normal. GDS2642 was composed of 9 patients with UC and 4 people with normal. GDS3119 was composed of 21 patients with UC and 5 people with control. GDS4519 was composed of 10 pairs of twins patients with UC and 10 pairs of twins people with healthy. GDS4365 was composed of 30 patients with UC and 13 people with control. Furthermore, the mean value of different probes was taken as the final expression value of the gene, for multiple probes corresponding to the same chip.

### Rat experiment and TCM formula

#### Animals

Eighteen six-week-old male Wistar rats, weighing 200 g ± 20g, were purchased from Beijing Vital River Laboratory Animal Technology Co. Ltd (license: SCXK(Beijing)2016-0006). All animals were raised in the Barrier Environmental Animal Laboratory of Dongzhimen Hospital of Beijing University of Chinese Medicine (license: SYXK(Beijing)2015-0001) and maintained under the National Standards for Laboratory Animals of China (GB 14925-2010). Our study was carried out in compliance with the ARRIVE guidelines. This study was approved by the Ethics Committee of Laboratory Animals of Dongzhimen Hospital of Beijing University of Chinese Medicine (No.20-51). Animals were kept separately in an SPF laboratory, with the breeding environment: temperature 25 ± 1 °C, humidity 50 ± 10%, free to food and drinking water, 12-hour day and night alternation, as well as adaptable feeding for 5 days.

#### Drugs

Concentrate granules of Senna Folium were procured from Tcmages Pharmaceutical Co., Ltd (Beijing, China). Hydrocortisone sodium succinate was purchased from Tianjin biochem Pharmaceutical Co., Ltd (Tianjin, China) and dextran sulphate sodium (DSS) was purchased from MP Biomedicals (Santa Ana, USA). The JFFZLZ granules were a compound preparation of Chinese herbal medicine, including Herba Schizonepetae (JingJie, Anguo City, Baoding City, Hebei Province, China, 21030321, 12 g), Radix Saposhnikoviae (FangFeng, Linxi County, Chifeng City, Inner Mongolia Autonomous Region, China, 21029071, 12 g), Radix Aconiti Lateralis Preparata (FuZi, Jiangyou City, Mianyang City, Sichuan Province, China, 21041201, 10 g), Radix Ginseng (RenShen, Fusong County, Baishan City, Jilin Province, China, 22001761, 10 g), Rhizoma Zingiberis (GanJiang, Luoping County, Qujing City, Yunnan Province, China, 21030521, 10 g), Radix Glycyrrhiizae (GanCao, Yuzhong County, Lanzhou City, Gansu Province, China, 21040401, 10 g), and Rhizoma Atractylodis Macrocephalae (BaiZhu, Qiaocheng District, Bozhou City, Anhui Province, China, 21048621, 10 g), was also purchased from Tianjin biochem Pharmaceutical Co., Ltd (Tianjin, China).

#### Rat model

After five days of adaptive feeding, all the rats were fed with 2 ml/100g/d of 15% Sennae Folium decoction concentrate granule solution for 21 days; at the same time, they were given hydrocortisone succinate sodium with 0.15 ml/100g/d at a concentration of 5 mg/ml subcutaneously injection in the neck for 10 days. From the 22nd day, the rats were given 3% DSS solution and normal water alternately for a total of three cycles. In the first cycle, the DSS solution was administered for 5 days and then the normal drinking water for seven days; and the second cycle was the same as the first cycle of administration; while, in the third cycle, the rats were only given DSS for 8 days. If diarrhoea, bloody mucus, congestion, oedema, erosion, and ulcer appeared in the colonic mucosa, the model was established successfully. The whole establishment day of the chronic relapsing UC rat model was 53 days.

#### Groups and intervention

Rats (*n* = 18) were divided randomly into three groups: blank group (BG), model group (MG), and JFFZLZ group (JFFZLZG), with 6 rats in each group. In the BG and MG, rats were administered 0.9% saline solution *via* oral gavage. The JFFZLZG rats were administrated by 14.8 g/kg/d (two-fold of clinical equivalent dosages) JFFZLZ granule *via* oral gavage. The JFFZLZ granule was dissolved in distilled water and given to rats. Rats were treated by gavage at a dose of 1 ml/100g/d, whose drug administration period was 28 days. After the intervention, the proximal colon tissues (0-4 cm above the anus) were fixed with 4% paraformaldehyde, then been paraffin-embedded, dewaxed, rehydrated, and stained with Haematoxylin–Eosin staining (HE). Meanwhile, the distal colon tissues (4–8 cm above the anus) were put into the cryopreservation tubes and stored frozen at −80 °C.

### RT-qPCR

The total RNAs were extracted from the biopsy tissue of colonic mucosa using the RaPure Total RNA Mini Kit (Magen, CN) according to the manufacturer’s instructions. Total RNA reverse transcription to cDNA was performed with the All-in-One First-Strand Synthesis Master Mix (with dsDNase) (BioMed, CN). RT-qPCR was performed using the Real-time PCR Detection System (Agilent Technologies, USA) with the Taq SYBR® Green qPCR Premix (BioMed, CN). The relative abundance of mRNA was using GAPDH as an internal control gene. The primers were provided in Supplement Table 1.

### Western blot analysis

Rat colon tissues were lysed with potent RIPA buffer (Beyotinme, CN) adding a complete protease inhibitor cocktail (Epizyme, CN). The tissue homogenates were centrifuged at 12,000 g at 4 °C for 10 min, and the supernatant containing proteins was collected. Then, protein concentration was determined with a BCA kit (Epizyme, CN). The resulting supernatant was mixed with SDS-PAGE protein loading buffer (5X) (PPLYGEN, CN) and the mixture was placed in a metal bath at 100 °C for 5 min to denature the protein. Next, an aliquot of 30 μg protein from each sample was separated using 10% SDS-PAGE (Epizyme, CN) and transferred to PVDF membranes. The PVDF membranes were incubated with primary antibodies against STAT3 (1:4000, 10253-2-AP, Proteintech, USA) and NF-kBp65 (1:1000, 66535-1-Ig, Proteintech, USA) at 4 °C overnight, and then incubated with secondary HRP-coupled antibodies (1:5000, SA00001-1, SA00001-2, Proteintech, USA) for 1 h at room temperature. The immunoreactivity was determined using enhanced chemiluminescence reagents (Biotech, CN) and analysed with Image J.

### ELISA

The expression of IL-6 and TNF‐α in rat colon tissues was determined by using ELISA Kit (RayBiotech, USA) according to the manufacturer’s instructions. The rat colon tissues were lysed with 2X Lysis Buffer (RayBiotech, USA) adding a complete protease inhibitor cocktail (Epizyme, CN). The tissue homogenates were centrifuged at 12,000 *g* at 4 °C for 10 min, and the supernatant containing proteins was collected. Then, protein concentration was determined with a BCA kit (Epizyme, CN). Then add 100 µL samples into the appropriate wells, and finally detected the OD value at 450 nm by the microplate reader. The concentration of target protein was calculated according to the standard curve and total protein concentration.

### Statistical analysis

Data for graphing was processed with GraphPad Prism 8.0 software. Statistical analyses were performed using the SPSS 25.0 statistical software package. One‐way analysis of variance (ANOVA) was used if the data as per normal distribution and homogeneity of variance, and the LSD test was used to assess the differences between the two groups. Otherwise, the Kruskal–Wallis H test was used. A *p*-value <0.05 was considered to indicate a statistically significant difference. Significance levels are *p < .05, **p < .01, ***p < .001, and ns: not significant.

## Results

### The interaction network between JFFZLZ targets and UC targets

**Figure 2.**
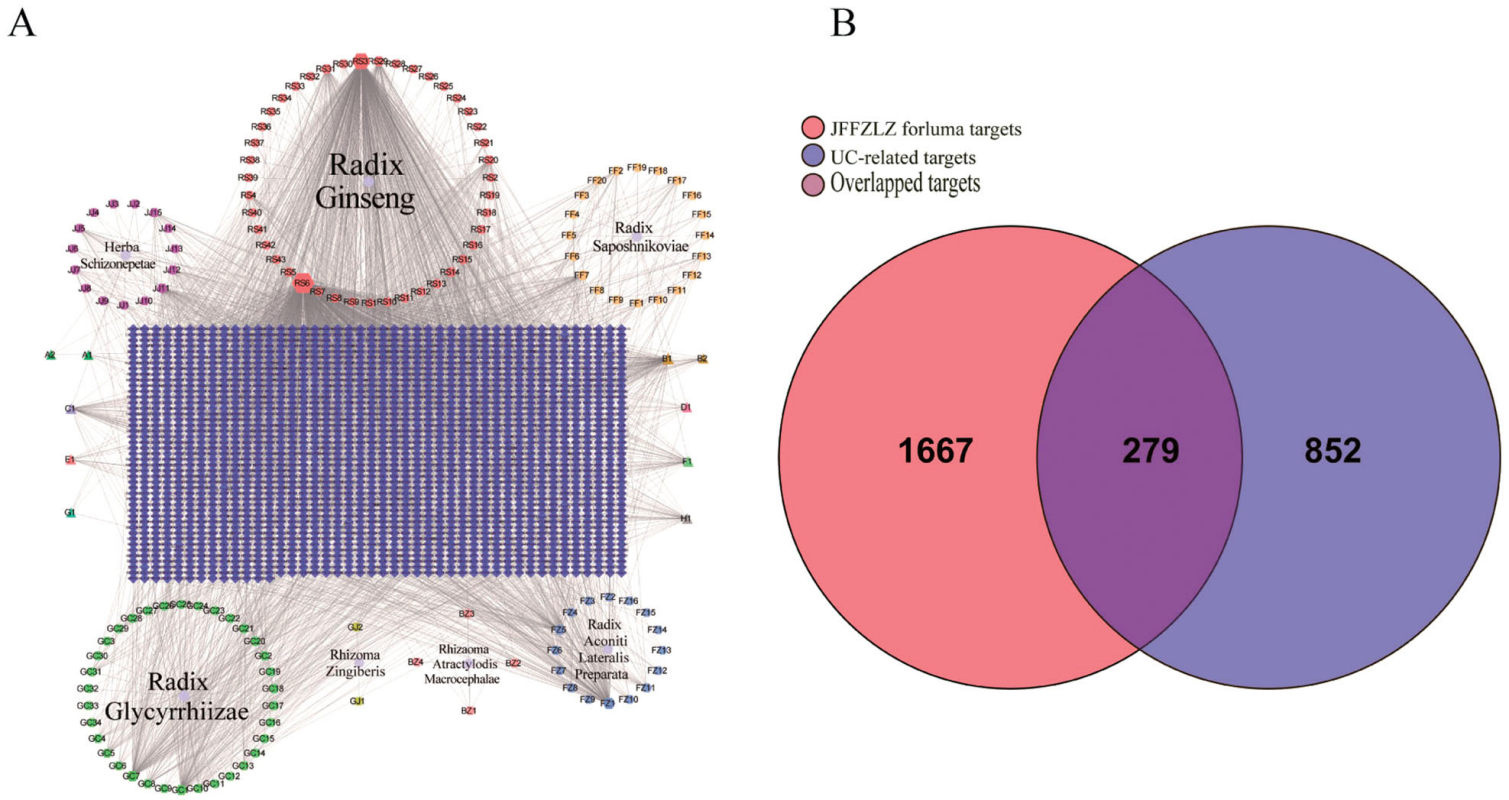
The interaction network between JFFZLZ targets and UC targets. (A) The constituent − target network. The blue rectangle means target; five circles, a pink square and a yellow vertical line mean constituent of JFFZLZ. Besides, the other’s letters mean repetitive ingredients. Meanwhile, A1, A2, B1, B2, C1, D1, E1, F1, G1, and H1 represents stigmasterol, folinic acid, quercetin, isoquercitrin, beta-sitosterol, daucosterol, deltoin, heptadecane, tridecanoic acid, and kaempferol respectively. (B) The OGEs between the JFFZLZ targets and UC targets.

First, we analysed the potential active constituents of seven herbs in the JFFZLZ and obtained 149 constituents from the TCMSP and TCMID databases after eliminating the redundancy (Supplement Table 2). According to the potential active constituents of JFFZLZ, there were 1,946 targets acquired from the TCMSP, TCMID, and SuperPred databases after removing the redundancy (Supplement Table 3). Then we constructed the interaction network between active constituents and targets of JFFZLZ by Cytoscape. Among the shared herb molecules, the proportion of beta-sitosterol was the highest constituent (6/7), followed by daucosterol (3/7). While stigmasterol, folinic acid, quercetin, isoquercitrin, deltoin, heptadecane, tridecanoic acid, and kaempferol were the lowest molecules (2/7) (Figure 2(A)). Next, a total of 1,131 UC targets were obtained from the DisGeNET, GeneCards, MalaCard, and TTD databases (Supplement Table 3). Finally, we obtained 279 overlapped genes (OGEs) between the JFFZLZ putative targets and UC targets (Figure 2(B)), the detailed information was shown in Supplement Table 3.

### Enrichment analysis for the OGEs

**Figure 3.**
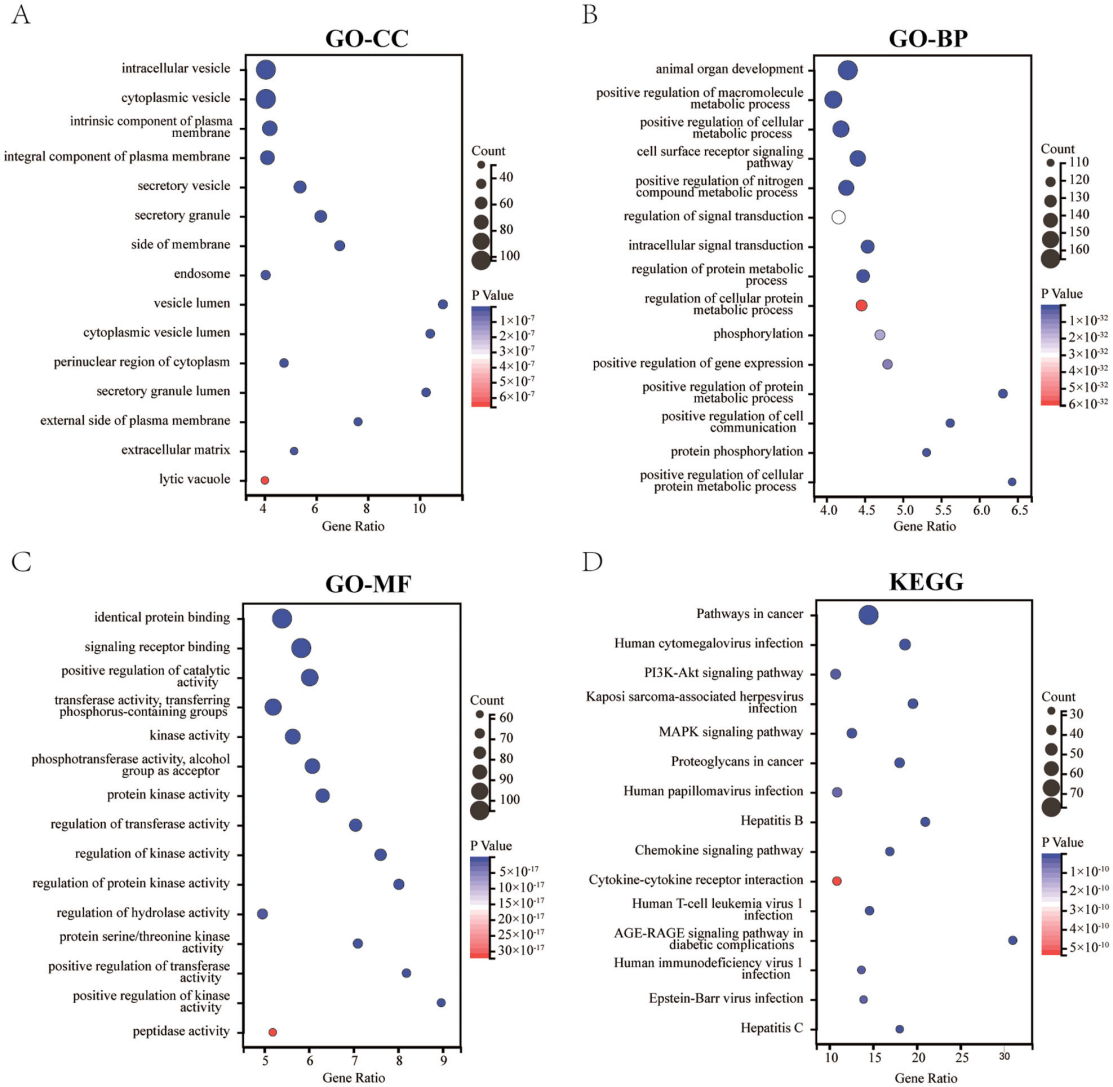
Enrichment analysis for the OGEs. (A) The 15 most enriched CC (*p* ≤ 0.01). (B) The 15 most enriched BP (*p* ≤ 0.01). (C) The 15 most enriched MF (*p* ≤ 0.01). (D) The 15 most enriched KEGG pathways (*p* ≤ 0.01).

To gain insights into the main biological functions of JFFZLZ against UC, the GO and KEGG pathway enrichment analysis for the OGEs between the JFFZLZ putative targets and UC targets were performed. The enrichment analysis results revealed that the GO-Cellular Component (CC) of OGEs were predominantly involved in the vesicles, plasma membranes, cytoplasm, and extracellular matrix (Figure 3(A)). The enrichment analysis results revealed that the GO-Biological Process (BP) of OGEs mainly involved organ development, signal transduction, phosphorylation, gene expression, and metabolic process (Figure 3(B)). The enrichment analysis results revealed that the GO-Molecular Function (MF) of OGEs were relevant to the binding functions, catalytic activity, a variety of transferase activities, kinase activities, hydrolase activity, and peptidase activity (Figure 3(C)). The KEGG enrichment analysis revealed that the OGEs were mostly involved in pathways in cancer, infection, hepatitis, PI3K-Akt signalling pathway, MAPK signalling pathway, Chemokine signalling pathway, Cytokine–cytokine receptor interaction, and AGE-RAGE signalling pathway in diabetic complications (Figure 3(D)). The details of GO and KEGG pathway enrichment analysis were highlighted in Supplement Table 4.

### Construction of PPI network between JFFZLZ targets and UC targets

**Figure 4.**
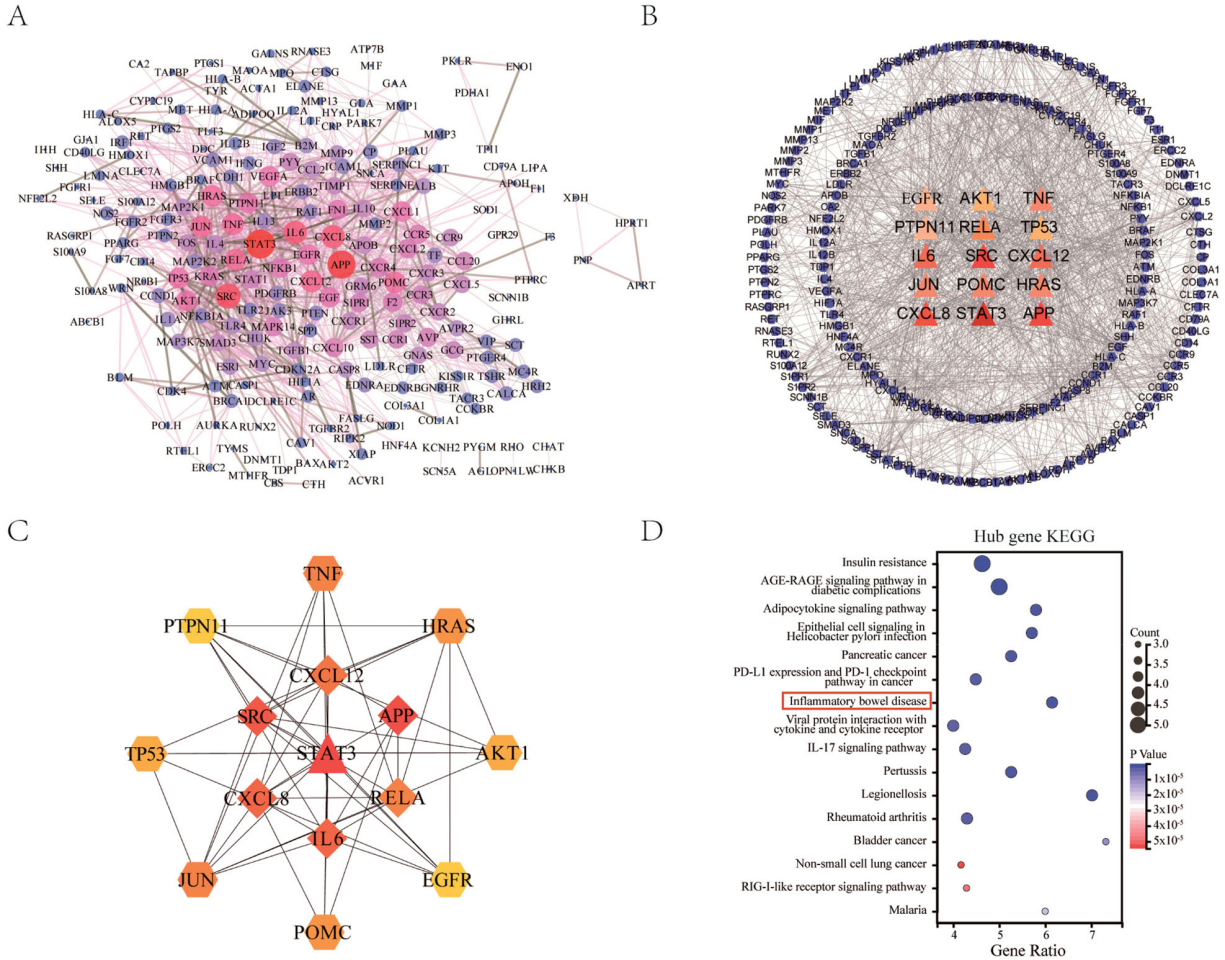
Construction of PPI network between JFFZLZ and UC, and screening of hub targets. (A) The PPI network between JFFZLZ and UC. Each node represents a protein and the connections represent the interaction between two proteins. A thicker connection represents a higher correlation. A bigger node means more important targets. (B) Hub targets. The central triangle means hub target. Colour depth means centricity in the network, the others refer to the remaining target of PPI. (C) The PPI network in hub targets. (D) KEGG enrichment analysis of the nine hub targets.

To better understand the relationship between different targets, the 279 OGEs were used to construct the PPI network through the STRING database and Cytoscape after eliminating non-interaction proteins. The PPI network contained 224 nodes and 1303 edges (Figure 4(A)), representing 1303 interactions among the 224 targets. Furthermore, after filtering with the network topological parameter degree, we identified the hub targets with a higher degree (score > 24) (Figure 4(B)). These hub targets included STAT3, APP, SRC, CXCL8, IL6, CXCL12, TNF, JUN, RELA, HRAS, POMC, TP53, AKT1, EGFR, and PTPN11. Also, the PPI network of targets was constructed, demonstrating the tight connections among the 15 targets (Figure 4(C)). However, among the hub targets, nine genes were induced genes related to UC, which we further analysed as core targets, including STAT3, CXCL8, IL6, CXCL12, TNF, RELA, TP53, and PTPN11. Then we performed a KEGG enrichment analysis of the nine hub targets. The results revealed that the hub targets were mainly involved in insulin resistance, diabetic complications, infection, cancer, inflammatory bowel disease, and IL-17 signalling pathways. Among these, the core pathway related to UC was inflammatory bowel disease (KEGG:05321), containing the hub targets of IL6, TNF, RELA, and STAT3 (Figure 4(D)).

### Molecular docking analysis for the core hub targets

**Figure 5.**
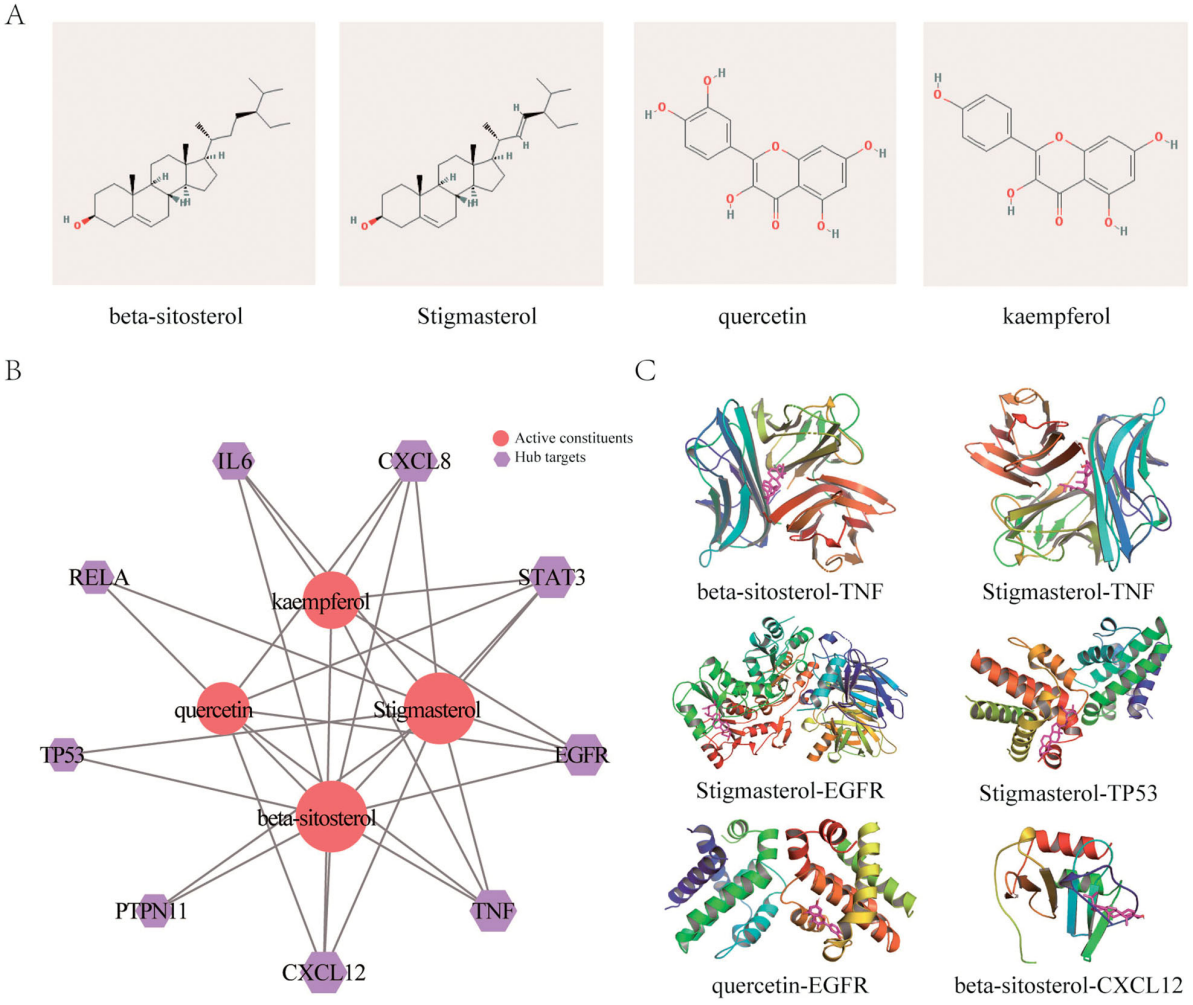
KEGG enrichment and molecular docking analysis for the core hub targets. (A) The 2D structure of the key constituents. (B) Interaction network between the key constituents and hub targets. The light red rounds represent active constituents of prediction, the purple hexagons represent hub targets. The size of the icon was set according to the degree of the network. (C) The 3D molecular docking structures of key compounds and key target genes.

To better understand the signal pathways and interactions between the constituents and target genes, we further constructed the molecular docking network between the active constituents and the hub targets. We first analysed the constituents in JFFZLZ according to the common degree and binding energy. As Table 1 showed, the key constituents we got were Stigmasterol (MOL000449), beta-sitosterol (MOL000359), quercetin (MOL000098), and kaempferol (MOL000422). Their 2D structures were shown in Figure 5(A). To further explore the interactions between the key constituents and hub target genes, we performed molecular docking analysis by AutoDockTools software (Figure 5(B)) and the docking scores of key constituents and hub targets were shown in Table 2. The 3D structures of six groups with the highest scores were shown in Figure 5(C).

**Table 1.** Common constituent in JFFZLZ.

Constituent	Herbs					
	*Herba Schizonepetae*	*Radix Saposhnikoviae*	*Radix Aconiti Lateralis Preparata*	*Radix Ginseng*	*Rhizoma Zingiberis*	*Radix Glycyrrhiizae*
	(JingJie)	(FangFeng)	(FuZi)	(RenShen)	(GanJiang)	(GanCao)
beta-sitosterol	√	√	√	√	√	√
Stigmasterol	√			√		
quercetin	√					√
kaempferol				√		√

**Table 2.** The docking scores of key constituents and hub targets.

Target	PDB ID	Stigmasterol	beta-sitosterol	Quercetin	Kaempferol
		(kcal/mol)	(kcal/mol)	(kcal/mol)	(kcal/mol)
STAT3	5ax3	−7.18	−7.04	−7.14	−6.61
CXCL8	1qe6	−6.72	−6.34	−5.29	−6.23
IL6	4zs7	−6.85	−5.35	−4.63	−5.32
CXCL12	2nwg	−7.32	−7.55	−6.35	−5.97
TNF	6op0	−9.35	−9.95	−6.97	−7.02
RELA	3qxy	−6.43	−5.26	−2.47	−4.57
TP53	1gzh	−7.61	−5.86	−4.7	−4.73
PTPN11	7r7i	−6.57	−7.46	−4.75	−4.43
EGFR	4qvx	−8.49	−7.4	−7.74	−6.96

### Expression of core hub targets in Colon tissues of UC patients

**Figure 6.**
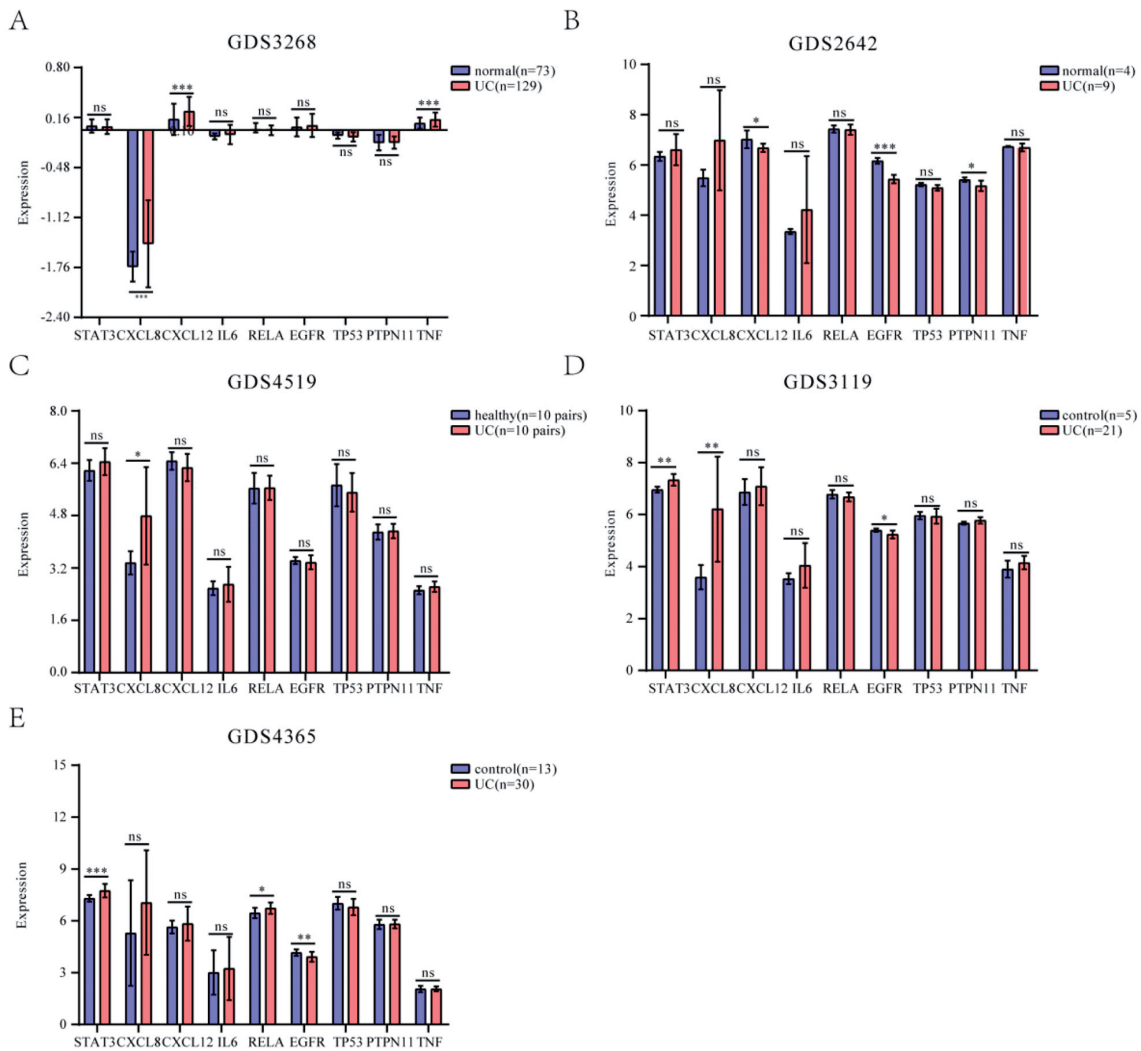
Expression of core hub targets in UC patients. (A-E) The core hub genes’s mRNA expression in normal and UC colon tissues in GEO datasets. Differences in means are considered statistically significant at *p* < 0.05. Significance levels are *p < 0.05, **p < 0.01, ***p < 0.001, and ns: not significant.

The GDS3268 dataset contained inflamed and un-inflamed colon epithelial biopsies of 129 UC patients from different anatomical locations of the gastrointestinal (GI) tract [25]. The GDS2642 dataset demonstrated the different patterns of gene expression in active and inactive areas of UC [26]. The GDS3119 dataset mainly studied the pro-inflammatory state in patients diagnosed with UC [27]. The GDS4519 dataset studied the relationship between microbiota and mucosal epithelium in the pathogenesis of inflammatory bowel disease based on twin pairs [28]. The GDS4365 dataset identified the molecular signature associated with UC remission [29]. We detected these nine genes’ expression in the above datasets. The results showed that some hub targets were significantly upregulated in UC patients compared to control, including STAT3, CXCL8, CXCL12, IL6, PTPN11, and TNF, while RELA, EGFR, and TP53 were downregulated (Figure 6(A–E)). These results indicated that the expression of these hub targets had possibly a bearing on the UC.

### JFFZLZ released the UC rats’ inflammatory response and downregulated the hub targets expression

**Figure 7.**
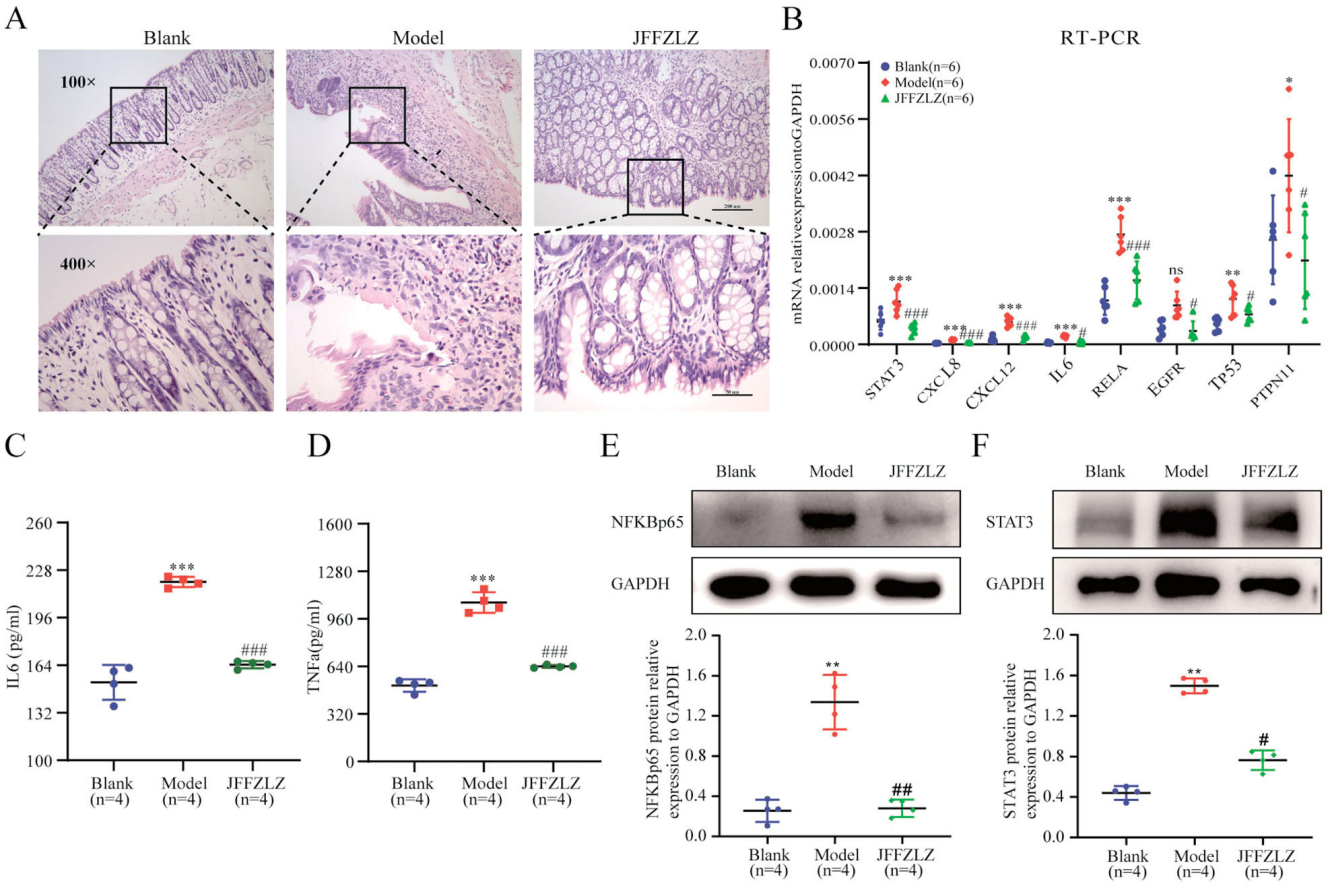
JFFZLZ released the UC rats’ inflammatory response and regulated the hub targets. (A) HE staining for rat colonic tissues. (B) The mRNA expression of the hub targets in rat colonic tissues, detected by RT-PCR and GAPDH as internal control gene. (C-D) The protein expression of IL6 (C) and TNFα (D) in rat colonic tissues, detected by ELISA. (E-F) The protein expression of NFKBp65 (E) and STAT3 (F), detected by WB, and GAPDH as internal control protein. Differences in means are considered statistically significant at *p* < 0.05. Compare to the Blank group, *p < 0.05, **p < 0.01, ***p < 0.001, and ***p < 0.0001. Compare to Model group, #*p* < 0.05, ##*p* < 0.01, and ###*p* < 0.001.

To further confirm the efficacy and mechanism of JFFZLZ against UC, we treated the UC rats with JFFZLZ and observed colon pathological changes by HE staining. The results showed significant colonic mucosal erosion and ulcers in the model group (MG) compared to the blank group (BG) (magnification, ×100), and significant inflammatory cell infiltration including neutrophils, eosinophils, plasma cells, and lymphocytes in the mucosal and submucosal layers (magnification, ×400). And, after JFFZLZ treatment, there were no apparent erosion and ulcers in the colonic mucosal, surface epitheliums and glands regenerated, and part of the glands appeared nodular(magnification, ×100). Meanwhile, the infiltration of inflammatory cells in the colon mucosal and submucosal layers significantly alleviated after JFFZLZ treatment compared to the MG rats (magnification, ×400) (Figure 7(A)). Then, we detected the mRNA expression of the hub targets in rat colon tissues by RT-qPCR. And the results demonstrated that the mRNA expression of the hub targets included STAT3, CXCL8, CXCL12, IL6, RELA, EGFR, TP53, and PTPN11 were all upregulated in the MG rats compared to the BG rats, and JFFZLZ treatment could downregulate these genes expression compared to MG rats (Figure 7(B)). On protein level, we detected the expression of IL-6 and TNF-α by ELISA, NFKBp65, and STAT3 by Western blot(WB) in rat colon tissues. These results indicated that the JFFZLZ’s therapeutic effect on UC relied on regulating the hub targets’ expression.

## Discussion

For the treatment of UC, glucocorticoids and mesalazine are the main options [30]. However, these medications have some flaws including osteoporosis, GI tract ulcers, immunocompetence, fever, diarrhoea, abdominal pain, and bloody stool [31,32]. In contrast, TCM treatment has unique advantages on UC, with better curative effects and lower prices [33]. Furthermore, during the clinical treatment, we found JFFZLZ had therapeutic effects on UC patients. Meanwhile, in this study, we also found the effect of JFFZLZ on UC in a rat model. Although JFFZLZ showed therapeutic efficacy on UC, the detailed mechanism was unclear and needed further research.

In this study, we first investigated and analysed the possible mechanisms of JFFZLZ against UC based on network pharmacology. We found that the key active constituents in JFFZLZ were stigmasterol, folinic acid, quercetin, isoquercitrin, beta-sitosterol, daucosterol, deltoin, heptadecane, tridecanoic acid, and kaempferol. These constituents were observed with inhibiting effects on colon inflammation. For instance, stigmasterol and beta-sitosterol had anti-inflammatory and antioxidant functions [34–36]; Likewise, daucosterol, folinic acid, quercetin, isoquercitrin, deltoin, heptadecane, tridecanoic acid, and kaempferol had similar antioxidant and anti-inflammatory effects [37–40]. These results suggested that JFFZLZ may function against UC *via* the interactions between these active constituents and their targets. Meanwhile, we also explored the targets of UC to understand its pathogenesis. We found that the inflammatory cytokines, signal transduction, autophagy, apoptosis, and cell metabolism accounted for the high proportion in UC. However, the detailed interaction information between key active constituents and targets needed further investigation.

Next, we constructed the GO-BP, GO-CC, GO-MF, and KEGG enrichment analysis. The result revealed that the GO-CCs were particularly related to the vesicles, cytoplasm, and extracellular matrix, both relating to the exosomes. The previous study has shown that exosomes could mediate the functional transfer of genetic materials between immune cells like macrophages, Treg cells, and neutrophils, which affects immune response [41,42]. The GO-BPs mainly involved signal transductions, phosphorylations, gene expression, and metabolic processes. And, the GO-MFs were relevant to the binding functions, kinase activities, and hydrolase activity. These results showed that JFFZLZ could influence the expression of key genes, the process of signal transduction, and the production of metabolites in the pathogenesis of UC. In some clinical studies, tofacitinib, a small-molecule Janus kinase inhibitor affecting the process of the JAK-STAT pathway, was shown to have more effective efficacy in patients with moderately to severely active UC [43–46]. The KEGG enrichment revealed that MAPK, Chemokine signalling pathway, and Cytokine-cytokine receptor interaction played a critical role. Several reports had shown that inhibiting the signal transmission process of the MAPK signal pathway could improve the symptoms of diarrhoea, mucous, and haematochezia in UC model animals [47–49]. These results suggested that the treatment efficacy of JJFZLZ against UC was achieved through integrating multi-constituent, multi-target, and multi-pathway.

Then, we identified the nine hub targets based on the PPI network and obtained the core KEGG signal pathway. In our study, the core targets were STAT3, CXCL8, IL6, CXCL12, TNF, RELA, TP53, and PTPN11. The core pathway related to UC was inflammatory bowel disease referring to IL6, TNF, RELA, and STAT3. Among the targets, CXCL8 and CXCL12 are chemokines, and STAT3, IL6, TNF, RELA belong to inflammation-related factors. As chemokines, CXCL8 and CXCL12 could recruit macrophages, T cells, B cells, dendritic cells, and other immune cells to induce inflammation [50,51]. Besides, IL6 and TNF could exacerbate the inflammatory response as pro-inflammatory cytokines [52]. Also, STAT3 and RELA are two critical factors in the inflammatory response [53]. Beyond these, EGFR, TP53, and PTPN11 are also the key genes in this study. It’s reported that EGFR could accelerate cellular proliferation, which was related to UC carcinogenesis [54]. TP53 is a critical gene during cell apoptosis which could affect UC’s inflammatory processes and intensity of symptoms [55]. PTPN11 was a cancer-related gene that induced macrophages to differentiate into type M1 *via* activation of RELA signalling, which promoted inflammatory response as well as induced carcinogenesis [56].

We further constructed the molecular docking network between the active constituents and nine hub targets. The result showed that Stigmasterol, beta-sitosterol, quercetin, and kaempferol had enhanced binding energy with nine targets. Among the above docking network, the beta-sitosterol-TNF was the highest docking network. To further verify the differential expression of the above genes in UC and normal subjects, we detected these genes’ expression in the GEO datasets and UC rat model. The treatment of JFFZLZ against UC was effective, and drugs were safe and had no toxicity (Supplement Fig. 1). As the result of the GEO dataset analysis, STAT3, CXCL8, CXCL12, IL6, PTPN11, and TNF all upregulated in UC patients, while RELA, EGFR, and TP53 were downregulated, compared to control people. At the same time, compared with the blank group, the nine hub genes were all upregulated in colon tissues of UC rats. And after JFFZLZ treatment, these genes’ expression was downregulated to normal levels. On protein level, the key proteins like IL-6, TNF-α, NFKBp65, and STAT3 of the inflammatory bowel disease pathway had the same change trend. These findings indicated that JFFZLZ could reduce the UC disease by reducing the expression of inflammatory factors and inhibiting the inflammatory pathways. Moreover, JFFZLZ may delay the carcinogenesis of UC, which needed to be further investigated and validated in clinical research.

To sum up, through the network pharmacology, data mining, and *in vivo* experiments, we found that the underlying mechanisms of JFFZLZ treatment on UC were related to cell oxidation, inflammatory response, signal transductions, gene expression, and metabolism. Meanwhile, it’s pivotal for the regulation of JFFZLZ on the hub targets. In the clinic, the combination of these targets could be used to evaluate the therapeutic efficacy for JFFZLZ and may also be applicable in other drugs. These findings could make several contributions to the treatment of UC at present and assist in providing a potential therapeutic strategy for UC in the future.

## Supplementary Material

Supplemental Material

Supplemental Material

Supplemental Material

Supplemental Material

Supplemental Material

## Data Availability

All the relevant data is provided within the paper and its supporting information files. The datasets analysed during the current study are available from the corresponding author on reasonable request. This work described was original research that has not been published previously and is not under consideration for publication elsewhere, in whole or in part.

## Abbreviations

UC: Ulcerative colitis
CRC: Colorectal cancer
TCM: Traditional Chinese medicine
JFFZLZ: JinFangFuZiLiZhong formula
OB: Oral bioavailability
DL: Drug-likeness
OGEs: Overlapped genes
PPI: Protein-protein interaction
GO: Gene Ontology
GO-CC: GO cellular component
GO-BP: GO biological process
GO-MF: GO molecular function
KEGG: Kyoto Encyclopaedia of Gene and Genome
GEO: Gene Expression Omnibus
DSS: Dextran sulphate sodium
BG: Blank group
MG: Model group
JFFZLZG: JFFZLZ group
HE: Hematoxylin-Eosin
RT-qPCR: Real-time quantitative polymerase chain reaction
WB: Western blot
ANOVA: One‐way analysis of variance
