# Supplementary figures and images for: Network pharmacology and in vivo experiment-based strategy to investigate mechanisms of JingFangFuZiLiZhong formula for ulcerative colitis
Source: Ann Med. 2022 Nov 16;54(1):3219–33. doi: 10.1080/07853890.2022.2095665 (PMC9673803; doi:10.1080/07853890.2022.2095665)

Supplement Fig.1

A

### Liver Function

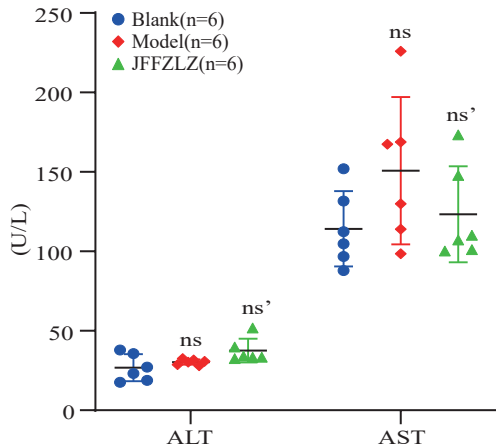

B

### Renal Function

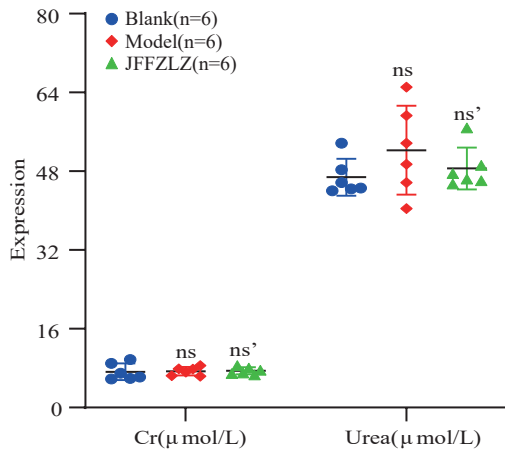

Supplement: Supplemental Material [file IANN_A_2095665_SM8198.pdf]
